# Low irrigation water minimizes the nitrate nitrogen losses without compromising the soil fertility, enzymatic activities and maize growth

**DOI:** 10.1186/s12870-022-03548-2

**Published:** 2022-04-01

**Authors:** Ihsan Muhammad, Ju Zhi Lv, Li Yang, Shakeel Ahmad, Saqib Farooq, Muhammad Zeeshan, Xun Bo Zhou

**Affiliations:** 1 Guangxi Colleges and Universities Key Laboratory of Crop Cultivation and Tillage, Agricultural College, Guangxi University, Nanning, 530004 China; 2 Maize Research Institute of Guangxi Academy of Agricultural Sciences, Nanning, 530007 China

**Keywords:** Nitrogen leaching, Irrigation water, N fertilizer, Biochemical processes, Enzyme, Maize growth

## Abstract

Nitrate nitrogen (NO_3_^−_^N) leaching increased with nitrogen (N) fertilization under high water supply to the field negatively affected the maize growth and performance. This study aimed to understand the mechanisms of NO_3_^−_^N leaching on a biochemical basis and its relationship with plant performance with 5 different doses (0, 200, 250, 300, 350 kg N ha^− 1^) of N fertilizers under low (60%; LW) and high (80%; HW) water holding capacity. Soil and plant enzymes were observed at different growth stages (V9, R1, R3, and R6) of the maize, whereas the leachates were collected at 10-days intervals from the sowing date. The LW had 10.15% lower NO_3_^−_^N leachate than HW, with correspondence increases in grain yield (25.57%), shoot (17.57%) and root (28.67%) dry matter. Irrespective of the irrigation water, RubisCo, glutamine synthase (GS), nitrate reductase (NR), nitrite reductase (NiR), and glutamate synthase (GOGAT) activities increased with increasing N fertilizer up to the V9 growth stage and decreased with approaching the maturity stage (R6) in maize. In HW irrigation, soil total N, GOGAT, soil nitrate (NO_3_^−_^N), leached nitrate (LNO_3_^−_^N), root N (RN), leaf N (LN) were positively correlated with N factors suggesting the higher losses of N through leaching (11.3%) compared to LW irrigation. However, the malondialdehyde (MDA), hydrogen peroxide (H_2_O_2_), superoxide (O_2_^−^), and proline were negatively correlated with the other enzymatic activities both under LW and HW irrigation. Thus, minimizing the NO_3_^−_^N leaching is possibly correlated with the LW and N300 combination without compromising the yield benefit and improving enzyme activities.

## Introduction

While protecting the environment quality, the global demand for higher food production is a serious challenge, especially in maize cropping [1]. Irrigation and N fertilizer are the two vital leading inputs affecting the agricultural cropping systems [2]. Nitrogen fertilizer is one of the key elements influencing crop growth. High water and fertilizer inputs are commonly seen to achieve high yields [3]. However, excessive fertilization and poorly planned irrigation systems are common methods causing soil environmental problems [4], resulting in a 20% N contribution to the environment due to farming [5]. Asia is the largest in N fertilizer consumption (58%), followed by the United States (22%), Europe (11%), and Africa (8%).

Regular N fertilization is needed to achieve optimum yields, especially in high-yield crops like maize (*Zea mays* L.). When fertilizer input exceeds crop requirements, contamination of water resources occurs [2, 6, 7]. The overuse of N increases the risks of nitrate leaching and causes water pollution, an international problem (Huang et al., 2017b). Nitrate pollution of groundwater is most common in areas with higher rainfall, light-textured soils, and high inorganic N fertilization [8, 9], and seriously damages the sustainability of food and agricultural soils [10, 11]. On the other side, the demand for food forced new agricultural practices to be developed, disturbing the N cycle in an agroecosystem [12]. There is a concern about optimizing the fertilization strategy because only a minute fraction of applied mineral fertilizer is absorbed by plants, and almost 30 to 50% of these nutrients can be lost in different ways [13].

Nitrogen in the form of nitrate is highly mobile in soil and is primarily influenced by soil water conditions [14]. However, the N leaching due to greater water availability degrades the local groundwater, which causes water pollution [15, 16]. High water is needed for improved crop growth and development; however, it causes potential N losses in the form of N leaching [6]. Thus, both N fertilization and water management are necessary for decreasing the potential nitrate losses without affecting the growth and performance of maize crops. The N balancing technique considers all N transformation activities in the experimental field, including urea volatilization, hydrolysis, mineralization, nitrification, initial soil N, plant N uptake, residual soil N, and N leaching [17, 18]. Consequently, both scientific and public attention has recently concentrated on preserving water against contamination caused by mineral N from different agricultural practices [19].

The maize crop is growing globally due to its multiple applications in the food, feed, and industrial sectors. Average maize yields in China increased from 1 ton (1949) to 6 tons ha^− 1^ (2013), demonstrating a 1633% increase in total maize production [20]. In China, maize was grown on more than 36 million hectares in 2013, with greater production than any other crop [21] especially, in North China Plain, which contributed about 39% of total production. Most farmers use their experience in fertilization and the economic situation rather than assessing the potential consequences of their decisions [22]. Ju et al. [23] stated that some farmers apply 500 to 600 kg N ha^− 1^ for better maize and wheat yield in crop rotation, with 60% application to maize. In North China Plain region, researchers have discovered that the average N application for the winter wheat-summer maize crop rotation system has increased fivefold in the last 30 years [6, 24], and thus caused 70% losses to the environment. Therefore, the N fertilization and irrigation optimization is the need of the day for a possible increase in N-use efficiency, reducing NO_3_^−_^N losses through leaching (8.43 kg N ha^− 1^ for 142 kg N ha^− 1^ fertilization) under 60 cm soil depth [2], and developing strategic management practices for increasing maize crop productivity and sustaining the environment [2, 25]. Therefore, this study was designed to investigate the variation in N transport and leaching with varying N fertilization levels under two irrigation levels (60 and 80% of field capacity) in maize crops. The objectives of this study were; (1) to assess the feasibility of growing maize at different irrigation and N levels, and the impact of this approach on soil physicochemical properties, grain yield, plant N content, and mineral N leaching, (2) to determine the effects of irrigation levels coupled with synthetic N fertilizer on soil N accumulation, and to develop a practical N management scheduling approach that integrates local factors like soil moisture, maize water use, and rainfall, and (3) to quantify the impact of higher N application rate on nitrate leaching under low and high irrigation water, and its relationship with soil plant N metabolism enzymes and maize growth.

## Material and methods

### Experimental design

A pot trial for 135 days in a greenhouse was carried out at Guangxi University (22°50′ 24.6“ N, 108°17’ 2.25” E) in the subtropical region of China. The average annual temperature and rainfall are 21.8 °C and 1298 mm, respectively, and the difference is 227 mm of precipitation between the driest and wettest months. The pots were filled with the soil of clay loam soil texture (according to Chinese Soil Taxonomy), with a pH of 5.6, a field capacity of 44%, soil bulk density of 1.40 g cm^− 3^, soil organic matter of 20 g kg^− 1^, and available N, phosphorus, and potassium of 127 mg kg^− 1^, 40 mg kg^− 1^, and 126 mg kg^− 1^, respectively. Five seeds of maize hybrid (Zhengda 619) were planted per pot (length of 32.5 cm and height of 29 cm) on September 28, 2020. The Zhengda 619 is the most commonly grown cultivated variety in the subtropical areas of China. The seeds were obtained from the CP seed industry Yunnan Zhengda seed Co. Ltd., China. The selected seeds permission was granted from the respective authority. The research was conducted according to ethical guidelines complied with relevant institutional, national, and international guidelines and legislation. Before sowing, basal fertilizers like phosphorus (P_2_O_5_) and potash (K_2_O) at the rate of 100 kg ha^− 1^ each, and half of the respective N fertilizers (as urea) were mixed with soil in a pot. However, the remaining half of the N dose was applied as a top dressing at the nine-leaf stage (V9).

The experiment consisted of two factors (i.e, irrigation and N treatments) conducted in control conditions. A 2 × 5 factorial experiment using four replication was carried out in a completely randomized design. The irrigation levels were (1) high irrigation 80% water holding capacity (HW) and (2) low irrigation 60% water holding capacity (LW) relative to the field capacity of the soil. The five N treatments were 0, 200, 250, 300, and 350 kg N ha^− 1^ application represented as control, N200, N250, N300, and N350, respectively. The pot positions were changed at a 10 day interval during irrigation inside the greenhouse to reduce the temperature effect.

Plants were irrigated with tap water during the entire growth period, and both HW and LW water levels were maintained. Micro-tensiometers were used to measure the water of the soil in each pot (Nanjing Institute of Soil Science, Chinese Academy of Sciences). The soil moisture content was corrected at five-day intervals by weighting and adding additional water to maintain the required HW and LW water contents. In addition, six tiny holes were made at the bottom of each pot used to collet leachate in plastic trays placed below the pots. After each irrigation, a syringe was used to collect the leachate from the plastic plate and store it at 4 °C for lab examination.

### Experimental analytical procedures

The full expended leaves at four different growth stages (V9, R1, R3, and R6), were collected for measuring the N metabolism enzyme activities. The activities of nitrate reductase (NR; D799304-0100), nitrite reductase (NiR; D799133-0100), glutamine synthetize (GS; D799578-0100), glutamate synthase (GOGAT; D799302-0100), RubisCo (D799834-0100), and glutamate dehydrogenase (GDH; D799834-0100) were determined using the plant enzyme kit from Sangon Biotech Co. Ltd. (Beijing, China) following the appropriate manual supplied with the kit. Similarly, the total N contestants in leaves, stems, roots, and grains of maize were determined following the Kjeldahl procedure.

The NO_3_^−_^N in the leached water was determined after the first (sowing time) and second dose (V9) of N application. The leachates collected from a plastic tray (1 L) every 10 days were stored at 4 °C in a 50 ml polyethylene tube until nitrate analysis. The UV-spectrophotometer was used to measure the nitrate contents in the leached water [26]. At the end of the experiment, the homogenized soil sample was taken from each pot. Roots and other debris were removed and half of the soil samples were air dried, sieved and used for the determination of soil total N using the Kjeldahl procedure [27]) and organic carbon (K_2_Cr_2_O_2_ extraction method). While half of the soil samples were kept in a refrigerator at − 80 °C until the determination of soil NH_4_^+_^N and NO_3_^−_^N as per the procedure of KCl extraction method proposed by Paramasivam and Alva [28]. The N use efficiency was calculated by using the following formula.

$$ANUE\left( kg\ {kg}^{-1}\right)=\frac{\left[ grain\ weight\ \left( fertilized\ pot\right)- grain\ weight\ (control)\right]}{N\ fertilizer\ applied}$$

Whereas the ANUE is the agronomic N use efficiency.

The maize was harvested in April 2021 and thoroughly washed with tap water. The plants were separated into their components i.e., root, stem, leaves, stem, and kernel. Kernel yield and kernel per ear were recorded after threshing to determine grain yield. After that, the plant biomass was sun-dried for three days before being oven-dried for 72 h at 70 °C to evaluate the dry matter content.

### Statistical analysis

All the statistical analysis were performed using SPSS v 25 (IBM SPSS Statistics; Chicago, NY, USA). A mixed-model analysis of variance (ANOVA) was used to calculate the effects of N rates and irrigation levels on N metabolism enzyme activities, nitrate leachate, and soil nutrient content. The differences between the means were determined using Tukey’s test at *P* < 0.05. The Mantel test was used in R v.3.63 to evaluate the relationship between the N content (soil N, leachate and plant N), plant enzyme activities, and irrigation levels.

## Results

### Nitrogen metabolism enzyme

**Fig. 1:**
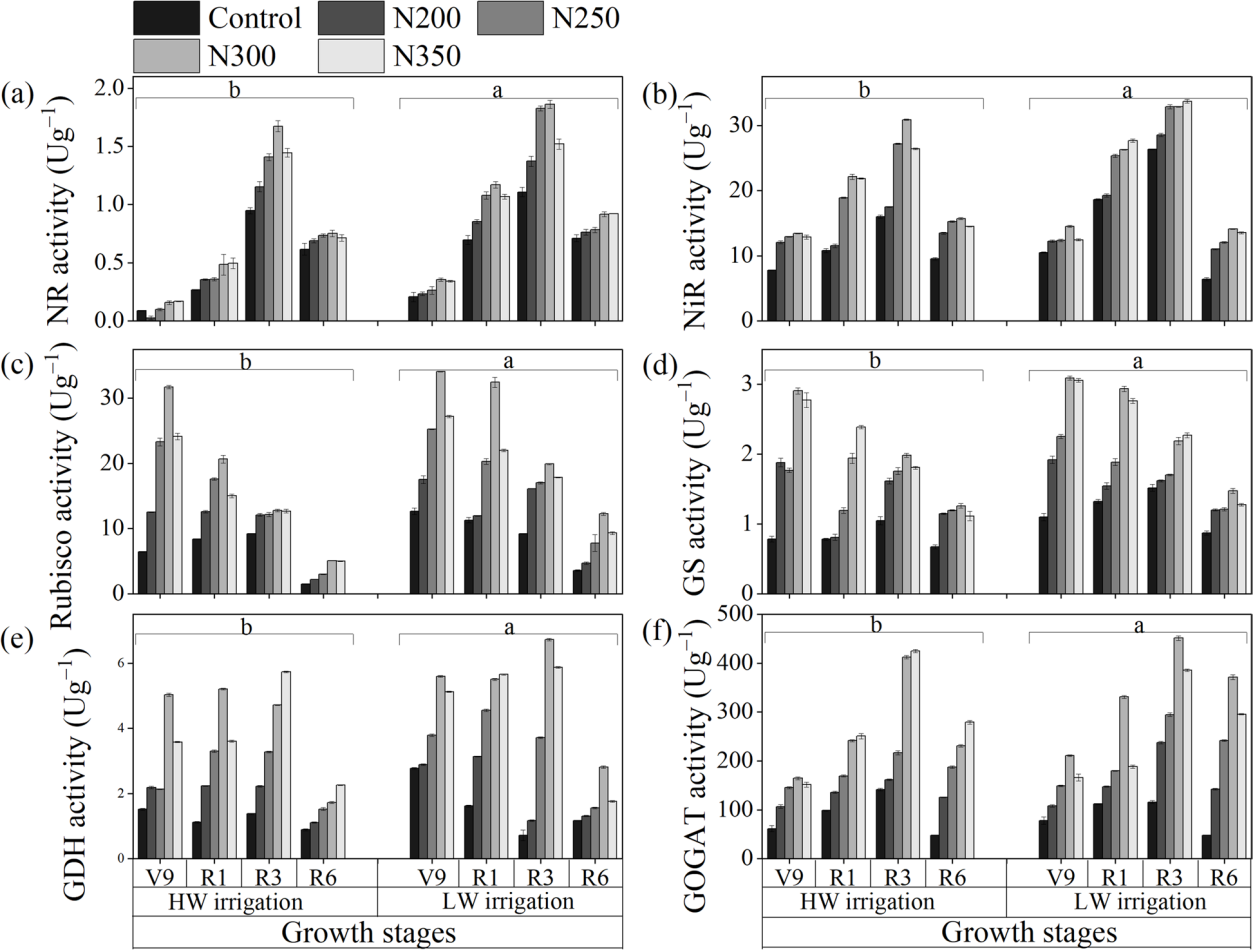
Effect of nitrogen dosages and irrigation regimes on the activities of nitrate reductase (NR), nitrite reductase (NiR), RubisCoRubisco, glutamine synthase (GS), glutamate dehydrogenase (GDH), and glutamate synthase (GOGAT). The bar represents the standard error. The nitrogen treatments control, N200, N250, N300 and N350 represent the application of nitrogen at the rate of 0, 200, 250, 300 and 350 kg N ha^− 1^. HW and LW represent the irrigation water at the rate of 80 and 60% of field capacity. The V9, R1, R3, and R6 stages represent the maize with 9 leaves, silking, milking, and maturity stages, respectively

**Fig. 2:**
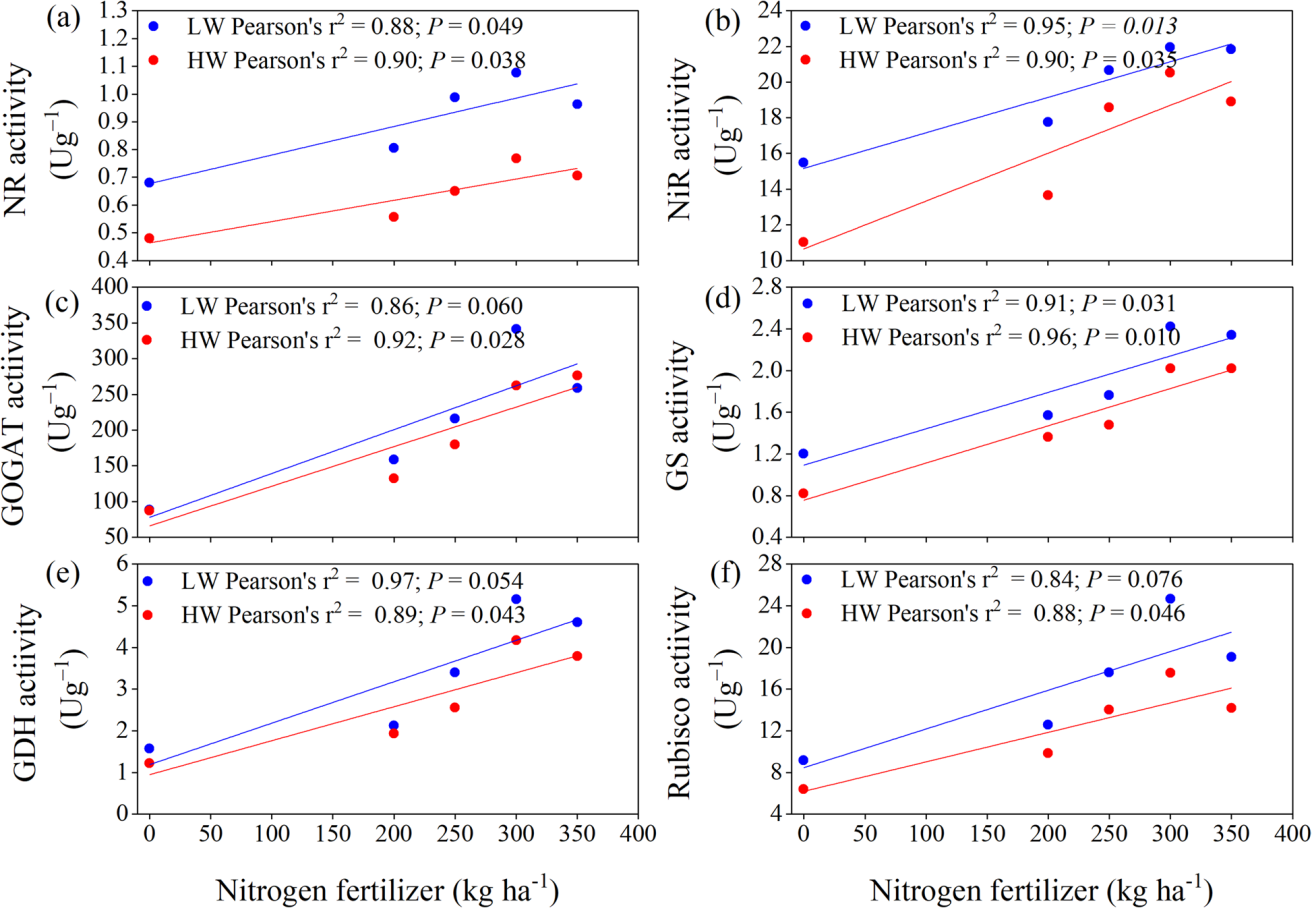
Effect of the interaction between nitrogen dosages and irrigation regimes on the activities of (**a**) nitrate reductase (NR) NR, (**b**) nitrite reductase (NiR) NiR, (**c**) RubisCoRubisco, (**d**) glutamine synthase (GS) GS, (**e**) glutamate dehydrogenase (GDH) GDH, and (**f**) glutamate synthase (GOGAT). The nitrogen treatments are control (0), 200, 250, 300 and 350 kg N ha^− 1^. The number of observation (*n* = 16) and HW and LW represent the irrigation water at the rate of 80 and 60% of field capacity

Remarkable differences in the N metabolism enzymes activities were noted in response to irrigation levels, N fertilizer treatments, and their interactions (Fig. 1) across the growth stages. The nitrate reductase (NR), NiR, and GOGAT activities increased with increasing N fertilizer up to N300 treatment at R3 growth (Milky stage) both under LW and HW irrigations (Fig. 1a, b, and d). However, the higher N fertilizer (N350) had decreased the enzyme activities. The growth stages positively affected NR, NiR, and GOGAT activities and showed increasing trends up to the R3 growth stage, but lower activities were found at the R6 stage. These enzyme activities were significantly higher in LW irrigation than in HW irrigation at all growth stages (*P* < 0.001). In contrast, RubisCo and GS activities were highest in plants at the V9 growth stage and decreased when approaching the maturity in maize plants (R6) under both LW and HW (Fig. 1c and d). The maximum RubisCo and GS activities were found for N300 treatment at the V9 stage under LW irrigation compared to HW irrigation. However, the lowest activities were observed in the control treatment at the R6 growth stage under LW and HW irrigation levels. The RubisCo and GOGAT activities in the maize leaves kept decreasing with crop maturity at all N treatments. An irregular increase in GDH activities (a key enzyme of glutamate pathways for biosynthesis) was detected for maize across the growth stages (Fig. 1e). However, at the R3 stage, the GDH activities were significantly higher in N300 and N350 treatments with LW and HW irrigation regimes, respectively. At the R3 stage with N300 treatment, the activities of NR, NiR, GDH, and GOGAT increased by 71.87, 50.60, 446.36, and 235.65%, respectively, compared to control treatment (*P* < 0.001). The regressions analysis showed that the N metabolism enzymes depend highly on irrigation water and N fertilization. In current study, the linear regression showed that NR (*r*^*2*^ = 0.88 and 0.90), NiR (*r*^*2*^ = 0.95 and 0.90), GOGAT (*r*^*2*^ = 0.86 and 0.92), GS (*r*^*2*^ = 0.91 and 0.96), GDH (*r*^*2*^ = 0.97 and 0.89), and RuBisCo (*r*^*2*^ = 0.84 and 0.88) activities were positively correlated to N fertilization under both LW and HW irrigation, respectively (Fig. 2). Furthermore, the linear regression confirmed that the enzyme activities were strongly and positively correlated to N fertilization.

### Soil nitrogen and carbon status

**Fig. 3:**
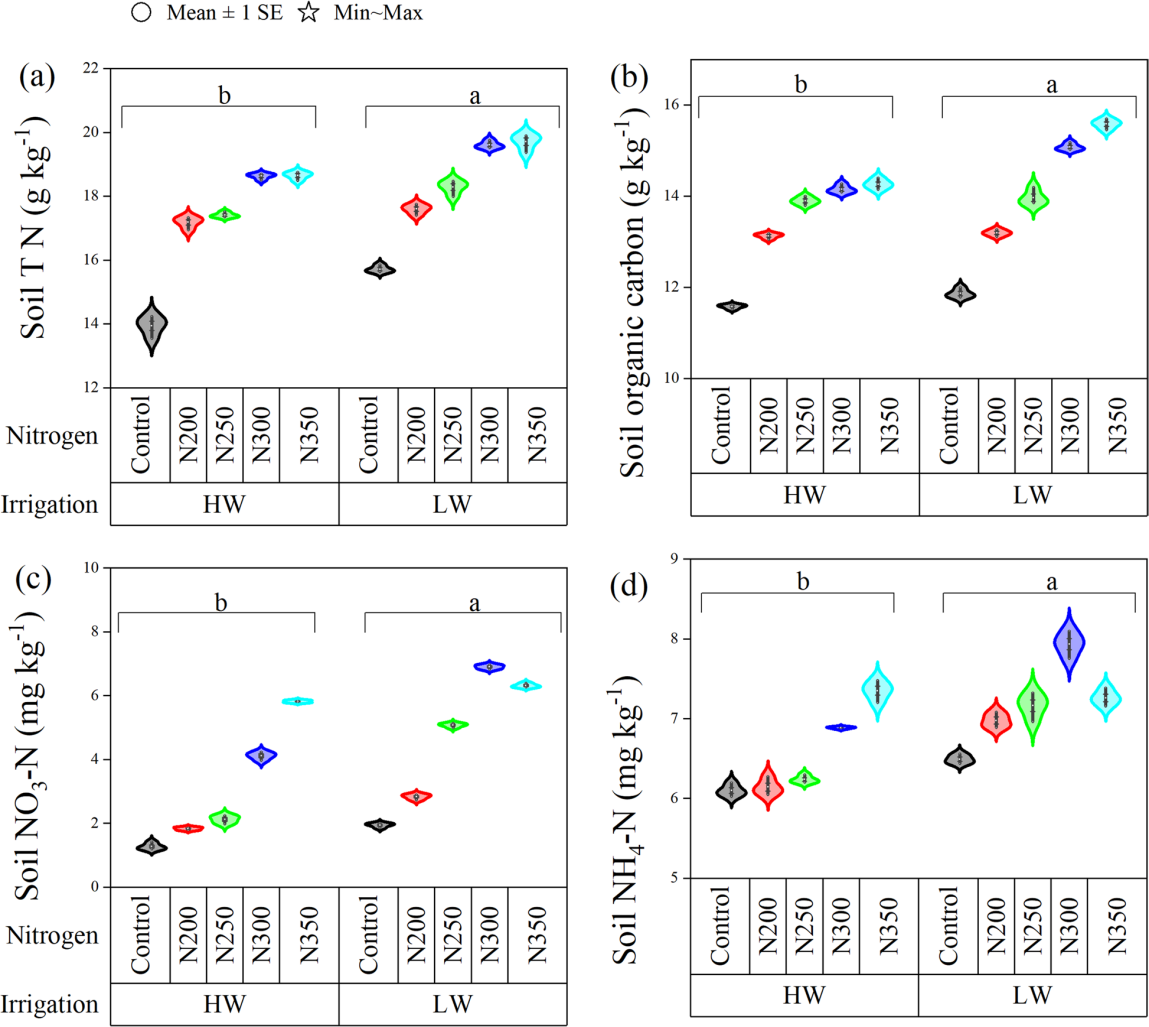
Effect of nitrogen dosages and irrigation regimes on soil total N, soil organic carbon, soil NO_3_^−_^N, and NH_4_^+_^N content. The bar represents the standard error (*n* = 4). The nitrogen treatments control, N200, N250, N300 and N350 represent the application of nitrogen at the rate of 0, 200, 250, 300 and 350 kg N ha^− 1^. HW and LW represent the irrigation water at the rate of 80 and 60% of field capacity. The V9, R1, R3, and R6 stages represent the maize with 9 leaves, silking, milking, and maturity stages, respectively

The soil NH_4_^+_^N, NO_3_^−_^N, total N, and soil organic carbon (SOC) contents were significantly affected by irrigation levels, N fertilizers, and their interaction (Fig. 3*P* < 0.01). Soil total N, SNH_4_^+_^N, SNO_3_^−_^N and SOC contents were increased by 29, 17.7, 243, and 24.7% in N300 treatment compared to N0, respectively (Fig. 3). The NH_4_^+_^N, NO_3_^−_^N, and SOC contents were highest in the N300 treatment, followed by the N350 treatment in LW irrigation. However, under HW irrigation, these parameters had resulted in greater values with N350 treatment than other N treatments. In contrast, the soil total N content was higher in the N350 treatment under both LW (19.7 g kg^− 1^) and HW (18.7 g kg^− 1^) irrigation levels as compared to the rest of N treatments (Fig. 3a). Our results indicate that the LW irrigation level significantly increased soil total N, SNH_4_^+_^N, SNO_3_^−_^N, and SOC content by 6, 9.5, 52, and 4% compared with HW irrigation.

### Leachate potential of nitrate

**Fig. 4:**
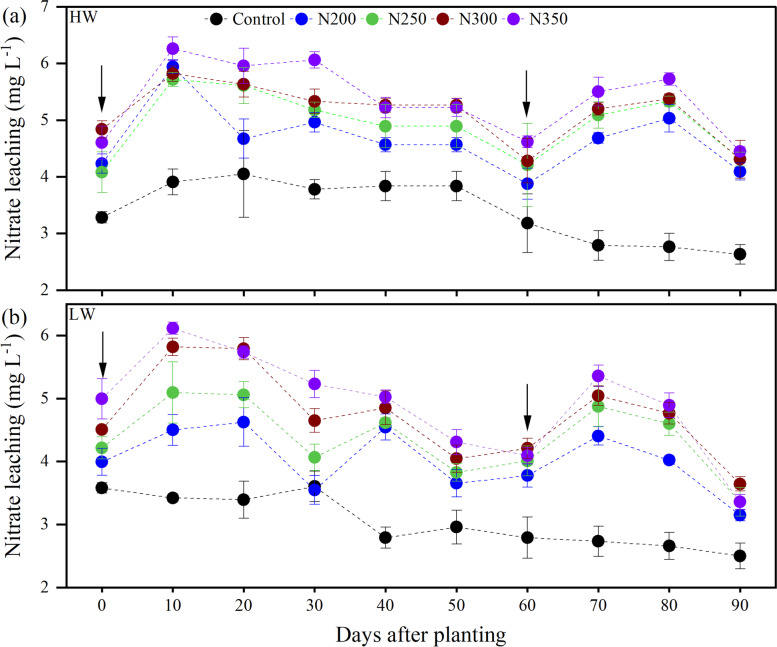
Effect of days, nitrogen dosages and irrigation regimes on soil NO_3_^−_^N leachate. HW and LW represent the irrigation water at the rate of 80 and 60% of field capacity. The bar represents the standard error (*n* = 4). The nitrogen treatments control, N200, N250, N300 and N350 represent the application of nitrogen at the rate of 0, 200, 250, 300 and 350 kg N ha^− 1^. HW and LW represent the irrigation water at the rate of 80 and 60% of field capacity

**Fig. 5:**
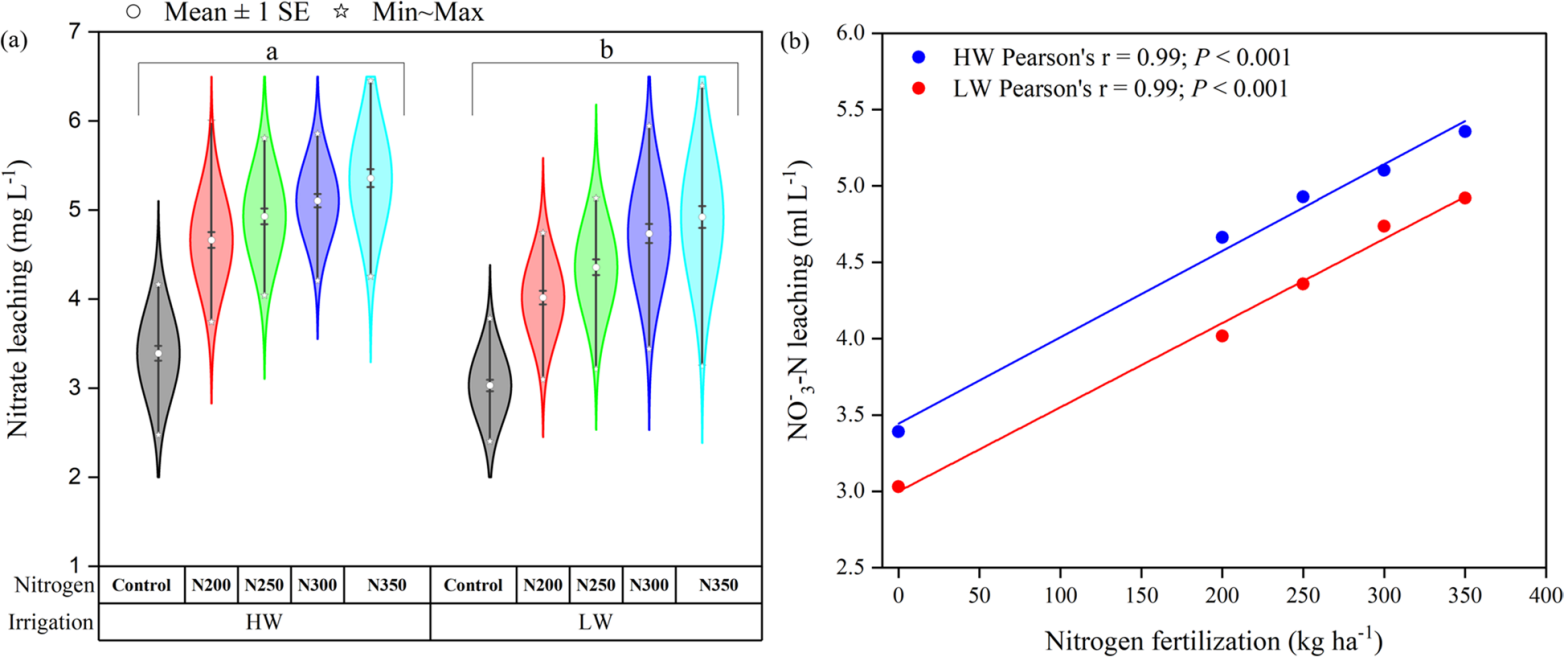
**a** Effect of nitrogen dosages and irrigation regimes on soil NO_3_^−_^N leachate, (**b**) the interaction between nitrogen dosages and irrigation regimes on NO_3_^−_^N leachate. The bar represents the standard error (*n* = 16). The nitrogen treatments control, N200, N250, N300 and N350 represent the application of nitrogen at the rate of 0, 200, 250, 300 and 350 kg N ha^− 1^. HW and LW represent the irrigation water at the rate of 80 and 60% of field capacity. The V9, R1, R3, and R6 stages represent the maize with 9 leaves, silking, milking, and maturity stages, respectively

The leachate potential of NO_3_^−_^N in response to irrigation levels and N fertilization across the growth stages showed variable responses to the treatments (Fig. 4). The NO_3_^−_^N content in leachate was maximum on 10 days after planting, concurrent with HW irrigation and the first dose of N fertilization (Fig. 4a). It was further noted that the maximum NO_3_^−_^N leaching was observed for N350, followed by N300 treatment, and the least for the control treatment. These results suggest that the NO_3_^−_^N leachate increased with increasing N fertilizer dosages under HW during the plant growing period compared to LW irrigation (Fig. 5a). Regardless of irrigation, the maximum leached NO_3_^−_^N content was observed for N350 treatment on day 10 after maize planting and gradually decreased with increasing days after planting up to 60 days (Fig. 4).

Furthermore, the leached NO_3_^−_^N increased again with 2nd dose of N fertilization on days 70 and 80 after maize planting. Average across fertilization, LW irrigation significantly decreased NO_3_^−_^N in leachate by 10.15% compared to HW irrigation (Fig. 5a). The maximum NO_3_^−_^N (5.36 mg L^− 1^) in leached water was observed in N350 treatment under HW irrigation, which was 8.84% higher than that of LW irrigation (Fig. 5a). The relationship between irrigation and N fertilizer showed that NO_3_^−_^N in leached water linearly increased with increasing N fertilizer dosages in both irrigation levels, but significantly lower leachate was observed under LW irrigation (*r* = 0.99; *P* < 0.001) compared to HW irrigation (*r* = 0.99; *P* < 0.001) as shown in Fig. 5B.

### Grain yield, dry matter and nitrogen contents

The irrigation and N fertilization varied the grain yield, shoot, and root dry matter of maize (Table 1). However, the interaction between these two factors were non-significantly difffent for root dry matter (*P* = 0.328). It was observed that N300 treatment under LW irrigation had higher shoot dry matter, root dry matter, and grain yield than the rest of the treatments. However, the shoot dry matter, root dry matter, and grain yield were highest in N350 treatment under HW irrigation than the other treatments. Average across fertilization, the LW irrigation significantly increased grain yield, shoot and root dry matter by 25.57, 17.44, and 28.66%, respectively, compared to HW irrigation. However, at the average across irrigations levels, the data showed that the N300 treatment significantly increased the grain yield by 82.13 and 2.06% compared to the control and N350 treatments, respectively.

**Table 1:** Effect of different water and N treatments on kernel yield, root and shoot dry matterr components of maize

N- fertilizer	Shoot dry matter (g)		Root dry matter (g)		Grain yield (g plant^−1^)		Nitrogen use efficiency (NUE)	
	HW	LW	HW	LW	HW	LW	HW	LW
Control	25.0 + 2.4 d	26.2 + 1.9 d	3.5 + 0.1 c	5.2 + 0.1 c	32.2 ± 0.6 d	27.9 ± 1.7 d	0.0	0.0
N200	34.3 + 1.5 c	38.4 + 2.2 c	5.6 + 0.2 b	6.7 + 0.2 b	4744.21 ± 1.32.2 abc	37.6 ± 1.8 c	17. 32 ± 0.5 a	14.75 ± 0.7 a
N250	36.7 + 4.1 bc	45.4 + 2.0 b	6.0 + 0.2 b	7.5 + 0.4 b	38.5 ± 0.2 bc	57.2 ± 1.0 b	9.65 ± 0.1 b	14.38 ± 0.3 a
N300	41.0 + 1.0 ab	54.9 + 1.8 a	6.8 + 0.4 a	9.2 + 0.5 a	39.4 ± 2.2 abbc	70.0 ± 1.2 a	6.88 ± 0.4 c	12.18 ± 0.2 b
N350	43.4 + 1.8 a	47.0 + 2.2 b	7.1 + 0.3 a	8.7 + 0.3 a	44.247.1 ± 2.21.3 a	60.1 ± 0.9 b	6.03 ± 0.3 c	7.7 ± 0.1 c

*Note* Means followed by different lowercase letters within each column indicate significant differences (*p* < 0.05) using LSD test. The nitrogen treatments control, N200, N250, N300, and N350 represent the application of nitrogen at the rate of 0, 200, 250, 300 and 350 kg ha^− 1^. HW and LW represent the irrigation water at a rate of 80 and 60% of field capacity

**Fig. 6:**
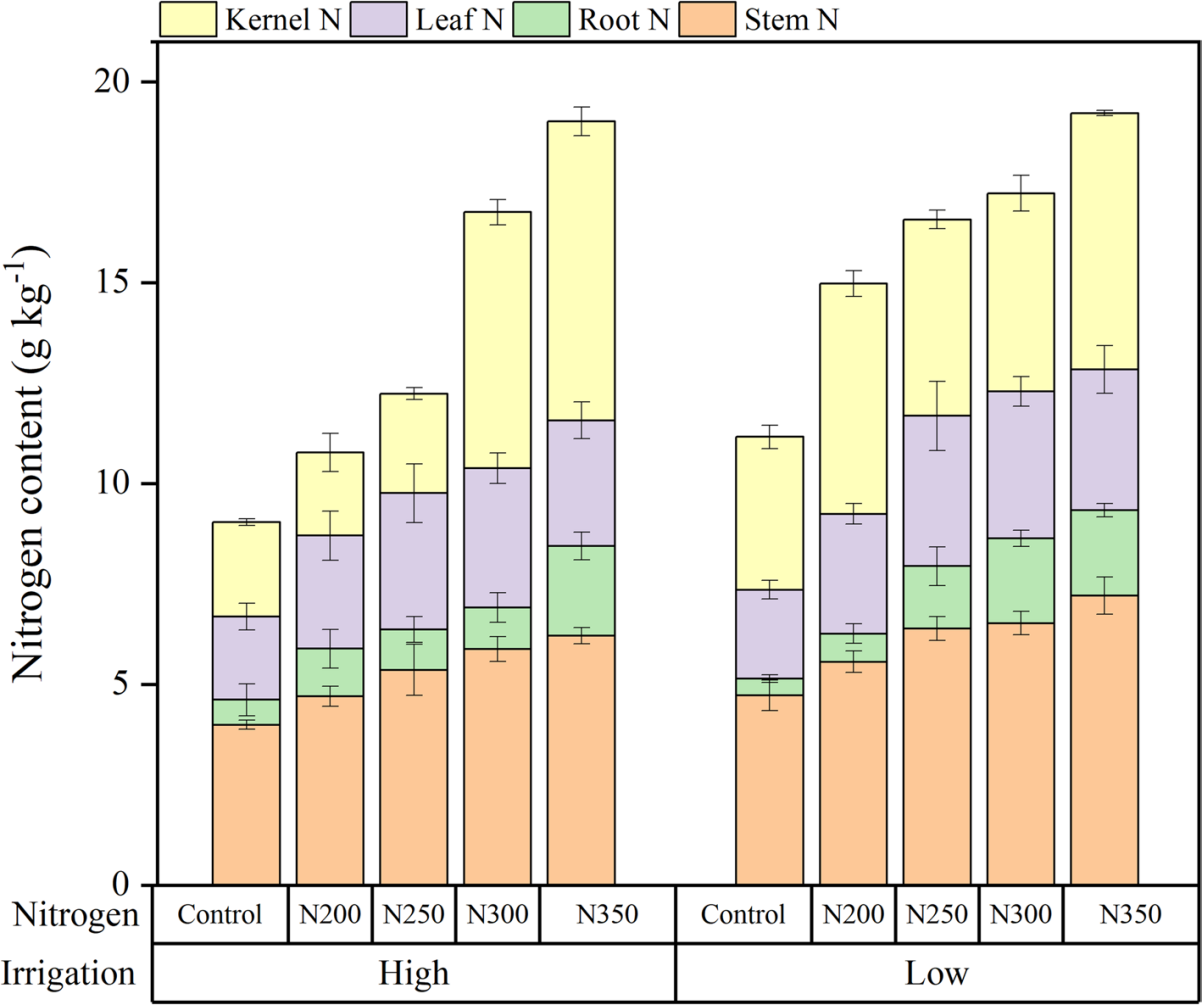
Effect of nitrogen dosages and irrigation regimes on kernel N, leaf N, root N and stem N content. The bar represents the standard error (*n* = 4). The nitrogen treatments control, N200, N250, N300 and N350 represent the application of nitrogen at the rate of 0, 200, 250, 300 and 350 kg ha^− 1^. HW and LW represent the irrigation water at the rate of 80 and 60% of field capacity. The V9, R1, R3, and R6 stages represent the maize with 9 leaves, silking, milking, and maturity stages, respectively

**Fig. 7:**
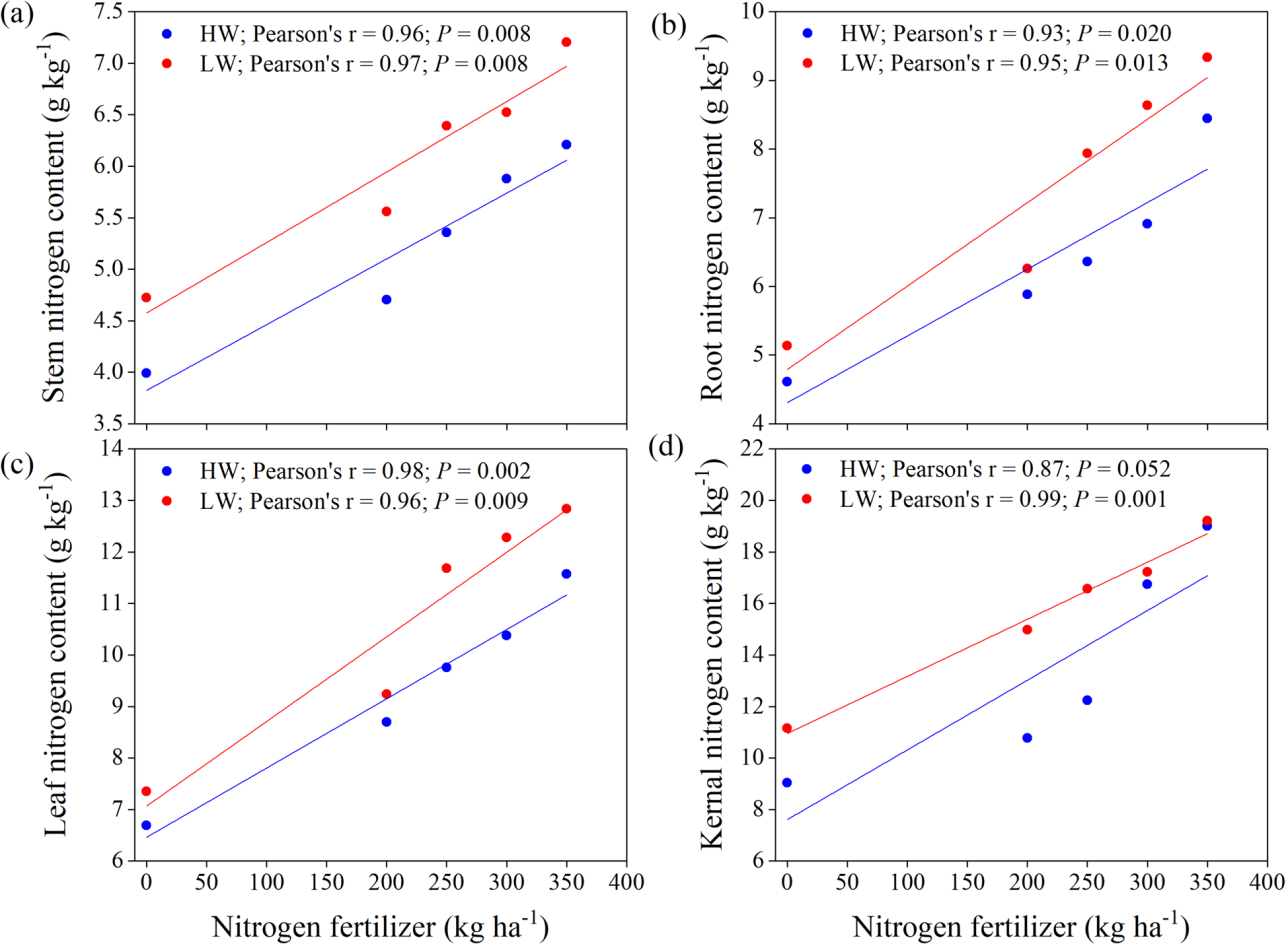
Effect of interaction between nitrogen dosages and irrigation regimes on kernel N, leaf N, root N and stem N content. The bar represents the standard error. The nitrogen treatments control, N200, N250, N300 and N350 represent the application of nitrogen at the rate of 0, 200, 250, 300 and 350 kg ha^− 1^. The number of observation (*n* = 4) and HW and LW represent the irrigation water at the rate of 80 and 60% of field capacity

Irrigation levels, N fertilizer, and their interaction significantly affected the kernel, leaf, root, and stem N contents (Fig. 6). Increasing the N fertilizer up to 300 kg N ha^− 1^ significantly increased leaf, root, and stem N contents, which was not statistically different from the N350 treatment (350 kg N ha^− 1^). However, the kernel N contents were significantly higher in N350 than other N fertilizer treatments. The plants N content in response to the irrigation levels showed that kernel, leaf, root, and stem N contents were significantly higher under LW irrigation compared to HW irrigation. Compared to LW irrigation, the kernel, leaf, root, and stem N content (averaged over N fertilizers) decreased by 14.33, 11.81, 13.65, and 14.02% in HW irrigation, respectively. The regression analysis showed that the stem (*r* = 0.98 (*P* < 0.01) and *r* = 0.99 (*P* < 0.01)), root (*r* = 0.98 (*P* < 0.01) and *r* = 0.99 (*P* < 0.01)), leaf (*r* = 0.98 (*P* < 0.01) and *r* = 0.96 (*P* < 0.05)), and kernel N (*r* = 0.98 (*P* < 0.01) and *r* = 0.96 (*P* < 0.01)) content linearly increased with increasing N fertilizers rate both under LW and HW irrigation regimes, respectively (Fig. 7).

### Nitrogen use efficiency

The present experiment revealed that different irrigation methods and N fertilizer significantly influenced the NUE in maize. The NUE was remarkably higher in LW compared to HW irrigation (Table 1). Our results showed that the NUE was significantly higher in N200 treatment compared to other N dosages. These results suggest that the NUE was 22.83% higher in LW irrigation compared to HW. Average across irrigation, N200 treatment resulted in 68.6 and 134.3% higher NUE than N300 and N350 treatments, respectively. These results indicate that NUE was significantly higher under LW irrigation with N200 treatment than other N treatments.

### Mantel test correlation analysis

**Fig. 8:**
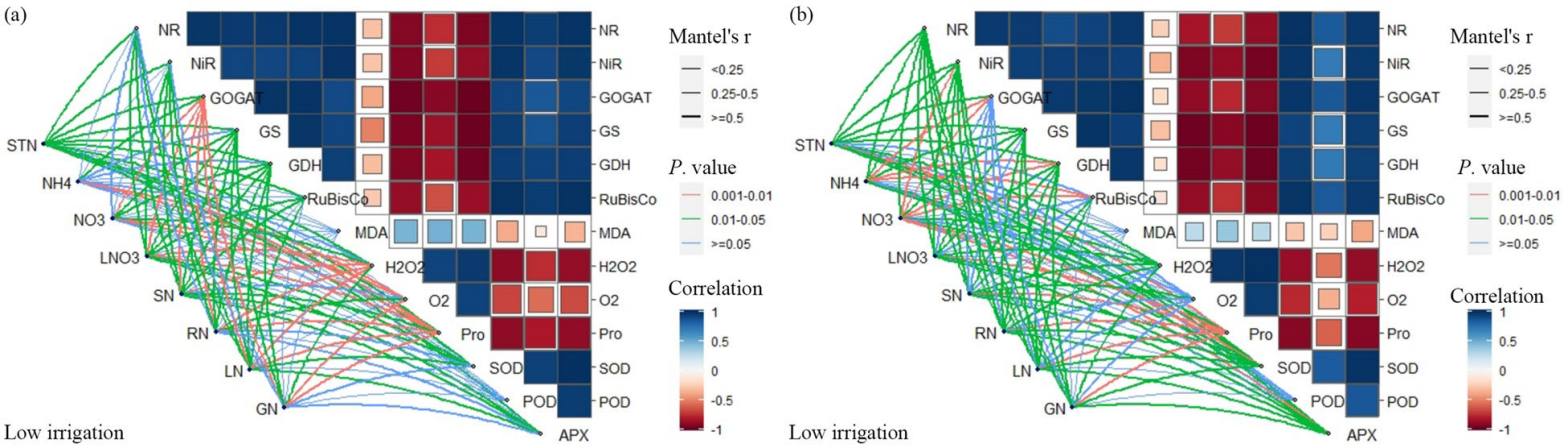
Correlation between nitrogen content (soil N, NO_3_^−_^N leachate, and plant N) and plant enzyme activities under two different irrigation regimes i.e. HW irrigation and LW irrigation. HW and LW represent the irrigation water at the rate of 80 and 60% of field capacity and GOGAT

The Mantel test was run to explain the relationship between the plant enzyme activities and the N factors of soil and plants (i.e, soil total N, mineral N, stem N, root N, leaf N, and grain N). The results revealed that the correlation strength for LW irrigation was higher than in HW irrigation (Fig. 8). The soil NH_4_^+_^N and NO_3_^−_^N of LW irrigation strongly positively correlated with NiR, GOGAT, GDH, and RubisCo activities. Similarly, the soil NO_3_^−_^N also strongly correlated with proline and APX activities (Fig. 8). All the N factors were negatively correlated with MDA, O_2_^−^, and POD activities, except SN, which had a lighter positive correlation with O_2_^−^ activity. In HW irrigation, STN is positively correlated with all plant enzyme activities except O_2_^−^. Likewise, the GOGAT activity was also positively correlated with all N factors, and the correlation between GOGAT and NO_3_^−_^N, LNO_3_^−_^N, SN, RN, LN, and GN were relatively stronger than others. Among the plant enzymes, all the enzymes were positively correlated with each other, except MDA, H_2_O_2_, O_2_^−^, and proline, which were negatively correlated with the other enzymatic activities under LW and HW irrigation regimes.

## Discussion

The high rainfall > 1600 mm and heavy N fertilization in the subtropical region’s agroecosystem is at risk due to greater NO_3_^−_^N leaching. In order to achieve high production without compromising the soil fertility and ecosystem, the farmers have to use more feasible irrigation water to avoid nitrate losses. The enhanced irrigation water and excessive N fertilization cause leaching into groundwater or through drainage ditches, which causes several environmental issues, including a decline of arable land, contamination of groundwater, eutrophication, and swamping wetlands downstream [6, 29, 30]. Our results showed that LW irrigation with N300 treatment significantly increased the grain yield, shoot and root dry matter compared to HW irrigation. The possible reason for this could be the lower N leaching in LW irrigation. These findings are consistent with previous researchers [6], who documented that NO_3_^−_^N leakage increases due to a huge amount of irrigation while using the same N fertilizer application rate. Irrigation and N fertilizers are the key limiting factors in plant growth. The soil total N, SOC, and mineral N mainly vary with irrigation and N fertilization [31, 32]. The results of the current study indicated that the soil total N, SOC, soil NH_4_^+_^N and NO_3_^−_^N content and distribution significantly differed under various irrigation levels and N fertilizer dosages (Fig. 3), signifying that the higher dosages of N (300 kg N ha^− 1^) resulted in higher soil total N, SOC and mineral N under LW irrigation (*P* < 0.05). These results are consistent with the findings of Wu et al. [31], who reported that soil nutrient content varied with irrigation and N fertilization. The high irrigation water might increase nutrient leaching and decrease the soil nutrient content [31].

The regression analysis described a linear relationship between plant N (stem, root, leaf, and kernel N) content and N fertilization (Fig. 7). These relationships were much stronger under LW irrigation than those of HW irrigation, possibly due to less nutrient losses through leaching and higher availability to plants in LW irrigation. Moreover, these outcomes demonstrated that the N fertilization should be adjusted to an optimum level (300 kg N ha^− 1^) under LW irrigation to minimize nutrient leaching and improve plant growth and yield. Previous studies demonstrated that the residual NO_3_^−_^N leaching increases abruptly when the N fertilization dosages increase [6, 33]. Similarly, applying N fertilizer without contemplating water availability or crop physiological demands may increase N losses through leaching and diminish plant N content [6]. Garcia et al. [34] reported that approximately 75% of the leaf N is allocated to the photosynthetic apparatus, and a strong relationship between photosynthetic rate and leaf N content was recorded. Several researchers reported that low and high N fertilization under water stress had lower tolerance than plants fertilized with optimum dosage [34, 35]. The GS activity decreased under water stress conditions. Thus, an increase in NH_4_^+_^N concentration and an increase in protein and chlorophyll loss in detached rice leaves [36] better explain these scenarios.

Nitrogenous substances and enzymes involved in N metabolism are significantly influenced by N fertilization and irrigation. An adequate level of nitrate accumulation facilitates the N metabolism in the cells, and excessive nitrate disrupts physiological processes [37]. We found that N300 treatment (averaged irrigation and stages) significantly increased NR, NiR, RubisCo, GS, GDH, and GOGAT activities compared to control. The current findings showed that lower N fertilization with HW irrigation could reduce plant N content, particularly in leaves, by negatively affecting N metabolism enzymes. Moreover, excessive N fertilization (350 kg N ha^− 1^) negatively influences plant enzyme activities, suggesting that a slow increase in these enzyme activities reduces the plant N content and yield. A study by Sun et al. [38] reported a positive correlation of soil enzymes with N fertilization, N availability, plant uptake and utilization at different plant growth stages.

The highest NR, NiR, GDH and GOGAT activities were found at the R3 growth stage with N300 treatment, while the RubisCo and GS activities were higher at the V9 growth stage. Natywa et al. [39] also demonstrated that the activity of enzymes is affected by N fertilization and crop growth stages [40–42]. On the other hand, improvements in soil fertility have been shown to increase the activity of NR and ammonia-assimilating enzymes such as GOGAT and GDH activities [43, 44]. High N doses did not increase enzyme activities. However, high N doses accumulate toxic substances, such as ammonia, which is harmful to plants and inhibits the growth of certain microbes, and ultimately decrease soil pH, which is crucial for enzyme performance [37, 45]. After flowering, the leaf is a chief source of N for grain, and the photosynthetic rate and RubisCo activity increases with increasing leaf N content [43, 46]. Moreover, these authors reported that water stress also causes metabolic imbalances resulting in amino acid accumulation, diminished ATP, and adversely affects NR activities [43, 46].

The NO_3_^−_^N leaching differed with varied N fertilization dosages and irrigation levels. The higher NO_3_^−_^N was found under HW irrigation and with a higher N dose (350 N kg ha^− 1^) at the early growing stage after the first 10 days of N fertilization application. Additionally, the NO_3_^−_^N losses (4.70 mg L^− 1^) through leaching were significantly higher under HW irrigation than the LW irrigation (4.22 mg L^− 1^). The increase in N fertilization and irrigation levels led to overall N losses through leaching [6]. These results were also supported by Gholamhoseini et al. [19], who reported that fertilizer N application from 0 to 450 kg N ha^− 1^ resulted in a ten and six folds increase in NO_3_^−_^N leaching in the HW and LW irrigation, respectively. In addition, they reported that 450 kg N ha^− 1^ increased the grain yield by 6% compared to 300 kg N ha^− 1^, but increased NO_3_^−_^N leaching by 67%. In contrast, other studies reported that higher N dosage does not increase crop yield but significantly increases NO_3_^−_^N leaching into groundwater [19, 47]. Even with LW irrigation, application of over 150 kg N ha^− 1^ did not significantly improve crop yield, but NO_3_^−_^N leaching was notably increased. Our findings indicated that NO_3_^−_^N leaching increased with increasing N fertilization and high irrigation water (Fig. 5a), which means that the N350 treatment under HW irrigation had the highest NO_3_^−_^N leaching compared to LW irrigation. The overall NUE was statistically higher in LW irrigation than HW; however, the N200 treatment under HW had significantly higher NUE than LW irrigation (Table 1). A previous study reported that irrigation water and N fertilization could improve the N absorption and utilization [48], play an important role in summer maize’s growth and development, boost N accumulation in various organs, and promote N accumulation in the ears [49].

In China, farmers apply about 500-600 kg N ha^− 1^ yr^− 1^ to maximize production in a maize-wheat rotation system. Thus, controlling the N application dosages is key to controlling nitrate concentrations in drainage waters, especially in the south subtropical regions of China. To minimize leaching losses, it is important to reduce the NO_3_^−_^N content in the soil, especially during the rainy season [6]. Land managers considered using optimal dosages of N fertilizer and the proper irrigation water for minimizing the leaching volume of NO_3_^−_^N. Tarkalson et al. [50] found that extremely heavy and deep seepage of surplus water and NO_3_^−_^N could be reduced using proper irrigation scheduling techniques [51]. Overall, we found a huge variation in NO_3_^−_^N leaching with different irrigation and N treatments, indicating that NO_3_^−_^N leaching increases with higher N fertilizer application and irrigation. Similarly, Gholamhoseini et al. [19] stated that grain yield was not significantly increased by applying more than 300 kg N ha^− 1^ in both irrigations, but NO_3_^−_^N leaching was dramatically increased.

## Conclusions

Higher N fertilization under limited irrigation supply positively affected maize crop yield, plant enzyme activities, plant N content, NO_3_^−_^N leachate and soil nutrient contents. The LW irrigation had 10.15% lower NO_3_^−_^N leachate than HW, with a correspondence increase in grain yield (25.57%), shoot (17.57%) and root (28.67%) dry matter. The RubisCo, GS, nitrate reductase, NiR, and GOGAT activities increased with increasing N fertilizer. In HW irrigation, soil total N, GOGAT, NO_3_^−_^N, LNO_3_^−_^N, RN, LN were positively correlated with N factors, suggesting the higher losses of N. However, the MDA, H_2_O_2_, O_2_, and proline were negatively correlated with the other enzymatic activities under LW and HW irrigation. Thus, minimizing the NO_3_^−_^N leaching is possible with the LW and N300 combination without compromising the yield benefit and improving enzyme activities. These findings suggest that optimizing the irrigation levels and N fertilization effectively enhances soil fertility, plant N content, enzyme activities and yield.

## Data Availability

The datasets used and/or analyzed during the current study are available from the corresponding author on reasonable request.
